# Physiological and neural synchrony in emotional and neutral stimulus processing: A study protocol

**DOI:** 10.3389/fpsyt.2023.1133760

**Published:** 2023-03-30

**Authors:** Maike Hollandt, Tim Kaiser, Heino Mohrmann, Jan Richter, Janine Wirkner

**Affiliations:** ^1^Department of Clinical Psychology and Psychotherapy, Institute for Psychology, University of Greifswald, Greifswald, Germany; ^2^Department of Experimental Psychopathology, Institute for Psychology, University of Hildesheim, Hildesheim, Germany

**Keywords:** attention, emotion processing, heart rate, ECG, skin conductance, SCL, EEG/ERP, synchrony

## Abstract

**Background:**

As psychotherapy involves at least two individuals, it is essential to include the interaction perspective research. During interaction, synchrony, i.e., the occurrence of simultaneous responses, can be observed at the physiological, neural, and behavioral level. Physiological responses include heart rate and electrodermal activity; neural markers can be measured using electroencephalogram. Emotionally arousing stimuli are allocated more attentional resources (motivated attention), which is reflected in physiological activation and brain potentials. Here we present a protocol for a pilot study implementing a new research methodology, and replication of the motivated attention to emotion effect in in dyads. There is evidence that higher synchrony is associated with more positive (therapeutic) relationships. Thus, the secondary outcome will be the association between physiological and neural synchrony and subjective ratings.

**Methods and design:**

Individuals (18−30 years) will participate in same-sex pairs in two experiments. In the first experiment (triadic interaction), both participants attentively watch unpleasant, neutral and pleasant pictures, and read/listen to standardized scripts (unpleasant, neutral, and pleasant, respectively) for the imagination task. In the second experiment, participants will read out three scripts (unpleasant, neutral, pleasant) to each other, followed by a joint imagination period. Stimuli will be presented in counterbalanced orders. After each picture and imagination, participants rate their subjective arousal and valence. In the beginning and in the end of the procedure, dyads rate their relationship, sympathy, and bonds (Working Alliance Inventory subscale). Heart rate, electrodermal activity and electroencephalogram will be continuously measured during both experiments using portable devices (EcgMove4 and EdaMove4, nine-channel B-Alert X-Series mobile-wireless EEG). Synchrony analyses will include the dual electroencephalography analysis pipeline, correlational analyses and Actor–Partner Interdependence Models.

**Discussion:**

The present study protocol provides an experimental approach to investigate interpersonal synchrony during emotion processing, allowing for the establishment of research methods in a pilot study, which can later be translated into real-life psychotherapy research. In the future, fundamental understanding of such mechanisms in dyadic interactions is essential in order to promote therapeutic relationships, and thus, treatment effectiveness and efficiency.

## 1. Introduction

To date, a main focus of psychotherapy research has been on randomized-controlled trials and meta-analyses to investigate the effectiveness of specific psychotherapy procedures and methods. However, robust data on predictors of response, and mediators of change are lacking, leaving the specific mechanisms of action of psychotherapy largely unclear (1, 2). A thorough understanding of the mechanisms of action and its biopsychosocial determinants allows for the identification of person characteristics that contribute to the effectiveness and efficiency of a specific psychotherapeutic intervention [including the neuroscience-based approach; e.g., (3)].

Since psychotherapy involves at least two persons, understanding the interaction between patient and therapist (e.g., relationship, synchrony) is likewise important to advance psychotherapy research. The interaction can be described as triadic, where therapist and patient focus on the same object (e.g., a work sheet or a phobic stimulus), and dyadic, which is the face-to-face interaction between therapist and patient (4). Synchrony refers to the occurrence of two or more people’s responses at the same time (literally); the notion of simultaneity, or similar expression, which can be more or less pronounced (4). For example, synchrony can be observed in body movements (5), voice tone (6), and physiological responses [for reviews, see Koole et al. and Mende et al. (7, 8)]. Physiological responses in synchrony research include heart rate (4, 9), skin conductance (10, 11), and brain activity (12). Brain activity of two or more people can be measured simultaneously using hyperscanning methods [e.g., (13)]. For hyperscanning, broader research from the field of developmental psychology on parent-child interactions exists [for reviews, see (14, 15)], but methods might translate well into psychotherapy research. For example, using functional near-infrared spectroscopy (fNIRS), synchronous brain activity has been observed in therapist-patient interactions, and synchrony was especially pronounced for experienced therapists, which was associated with better working alliance ratings (16, 17). Furthermore, neural synchronization has been found to predict learning outcomes in teaching contexts [for meta-analysis, see Zhang et al. (18)], leading to the assumption that synchrony might also play a crucial role in new (inhibitory) learning essential to psychotherapy success (19). However, data on hyperscanning in psychotherapy is still a white spot on the research agenda.

Preliminary research found that positive relationships (e.g., a good therapeutic alliance/bond as a prerequisite for psychotherapy) go along with higher synchrony between individuals (7, 20). Although positive correlations between therapeutic alliance and therapy outcomes are modest, they are a robust finding (21). Accordingly, interactional subjective and (neuro-) physiological data should be integrated into the holistic view of mechanism-based personalized psychotherapy research.

The aim of the present study is to implement the measurement methodology and analysis of interpersonal synchrony using paradigms for emotion induction already established in individual settings. These allow for the simulation of emotional and neutral interactions, as they may also occur in psychotherapy, under laboratory-controlled, experimental conditions and in healthy students.

Emotions can be induced *via* various stimuli. Standardized picture material [International Affective Picture System, IAPS; e.g., (22–24)] and scripts for mental imagery [e.g., (25–29)] are particularly suitable. In contrast to neutral information, emotional content is allocated more attentional resources, and, consequently, more in-depth processing [motivated attention; (30)]. These processes can be investigated using neural (electroencephalogram; EEG) and peripheral physiological correlates, e.g., electrocardiogram (ECG, including heart rate, heart rate variability) and electrodermal activity (EDA).

During picture viewing, a triphasic pattern of heart rate response is usually observed, including (1) initial deceleration (orienting response), (2) acceleration, and (3) secondary deceleration (30, 31). The present pilot study focuses on the first two observations. Unpleasant (and, less consistently, high arousing, in contrast to low arousing pleasant) pictures have been found to evoke more pronounced initial HR deceleration, whereas pleasant pictures provoked higher acceleration relative to unpleasant [neutral pictures ranging in between; (30, 32)]. In line with these findings, evidence from appetitive and aversive conditioning suggest, that initial bradycardia might be rather valence-independent, indicating stimulus novelty or significance (33, 34).

Viewing of emotionally arousing, relative to neutral pictures has been associated with higher skin conductance level (SCL), pointing to higher sympathetic arousal (29, 30, 32). In the EEG, and especially in event-related potentials (ERPs), elevated Late Positive Potentials (LPPs) for emotionally arousing, relative to neutral pictures, have been observed over centro-parietal electrodes, starting at about 400 ms post-stimulus onset and lasting for several 100 ms, likewise indicating motivated attention (23, 24, 35). Preceding the LPP, the early posterior negativity (EPN, 150−350 ms post-stimulus onset) is also reliably enhanced for emotionally arousing, relative to neutral stimuli (36).

Similar reaction patterns during picture viewing and imagery have been observed for heart rate and skin conductance [(37, 38); for summary, see Ji et al. (39)]. In contrast, during imagery, the LPP emotion effect might emerge over more posterior electrodes, compared to picture viewing (40). However, there are conflicting LPP results, and research on ERPs of emotional imagery is still sparse (40, 41).

## 2. Methods and analysis

### 2.1. Participants

Inclusion criteria for participation in the intended study will be German language (level B2 or higher), student status (University of Greifswald), and age between 18 and 30 years. Females will be required to use oral hormonal contraceptives to exclude menstrual cycle influences on emotion processing [e.g., (42, 43)].

Exclusion criteria will be non-Caucasian, current or past mental or neurological disorder, as well as acute illness and/or medication. Participants will be required to have normal or corrected-to-normal vision and hearing. Participants will receive course credit for participation. For controllability, only same-sex pairs with heterosexual orientation will be included.

### 2.2. Stimulus materials

#### 2.2.1. Picture stimuli

Sixty pictures (20 unpleasant, 20 neutral, 20 pleasant) have been selected from the International Affective Picture System [IAPS (22) and the EmoPics (44)] according to their normative valence (unpleasant: *M* = 2.4, SD = 0.63; neutral: *M* = 5.22, SD = 0.41; pleasant: *M* = 7.11, SD = 0.63; all *p*s < 0.001) and arousal ratings (unpleasant: *M* = 6.13, SD = 0.6; neutral: *M* = 2.87, SD = 0.33; pleasant: *M* = 5.97, SD = 0.79; unpleasant = pleasant >neutral, *p* < 0.001). Semantic categories include attack, mutilation, accident/disgust for unpleasant, neutral objects and people, and nature/buildings for neutral, and cute animals, babies/people, erotica, and sports/adventure for pleasant scenes, respectively, (see Supplementary material 1).

#### 2.2.2. Imagination scripts

Nine scripts (3 unpleasant, 3 neutral, 3 pleasant) have been selected from the Affective Norms for English Text [ANET; (26)], German translation (see Supplementary material 1).

### 2.3. Procedure

After arrival to the lab, participants will provide written informed consent to the study protocol approved by the ethics committee of the University Medicine Greifswald and sensors will be attached according the manufacturers’ manuals. If necessary, participants will get to know each other introducing themselves briefly (first name, age). Then, they will fill in the modified Working Alliance Inventory [WAI-SR, German; (45)] bonds-subscale (Items 3, 5, 7, 9, see Supplementary material 2) and rate their relationship and sympathy on a nine-point Likert scale (1 = “completely unknown/do not like at all” to 9 = “very well-known/like very much”; ratings are never revealed to the partner and participants have to sign a declaration of confidentiality). Data acquisition will take place in a sound-attenuated, dimly lit cabin. Following a 10 min baseline (rest, black screen), experiments A and B will be conducted in counterbalanced order. Finally, individuals will fill in the bonds-subscale (WAI-SR) and will be told the objectives of the experiment. Apart from the experimental interaction procedure, participants will be instructed neither to engage in conversation, nor to communicate *via* gesture or mimics.

#### 2.3.1. Experiment A—Triadic synchrony

Participant pairs will be seated in front of a 27-inch computer screen (*distance* = 1.5 m) and stimuli will be presented using Presentation Software (Version 23.1; Neurobehavioral Systems Inc., Berkeley, CA, USA).

##### 2.3.1.1. Picture viewing task

**FIGURE 1:**
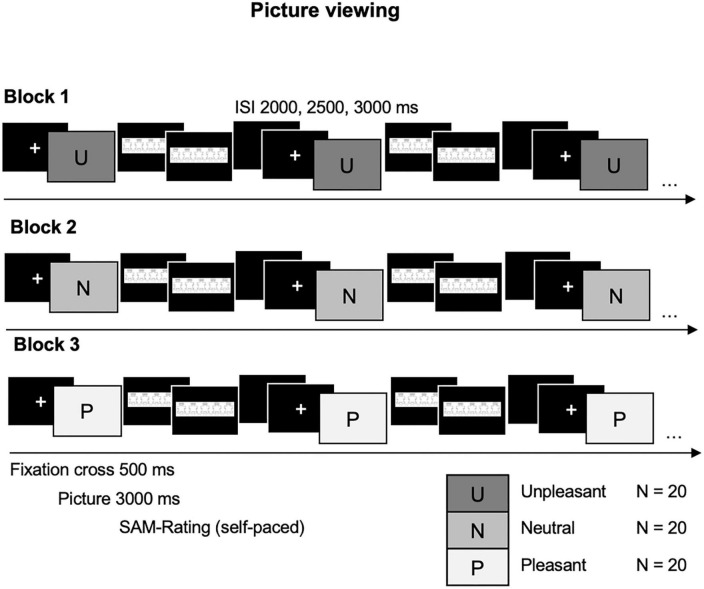
Picture viewing task. Blocked presentation of unpleasant (U), neutral (N), and pleasant (P) pictures for 3000 ms, following a 500 ms fixation cross; self-paced Self-Assessment Manikin (SAM) ratings follow each picture presentation. Variable inter-stimulus interval (ISI) of 2,500, 3,000, and 3,500 ms.

A block design has been chosen to avoid serial position effects for emotionally arousing and neutral pictures [(23); see Figure 1] Neutral (N), pleasant (P), and unpleasant (U) picture blocks will be presented in six orders randomly assigned to each participant pair (NPU, NUP, UPN, etc.). Pictures will be presented for 3,000 ms following a fixation cross of 500 ms (varying inter-stimulus interval 2,000, 2,500, and 3,000 ms). Individual self-paced Self-Assessment Manikin (SAM)-ratings for valence and arousal on a 9-point Likert scale (46) will follow each picture presentation.

##### 2.3.1.2. Imagination tasks

**FIGURE 2:**
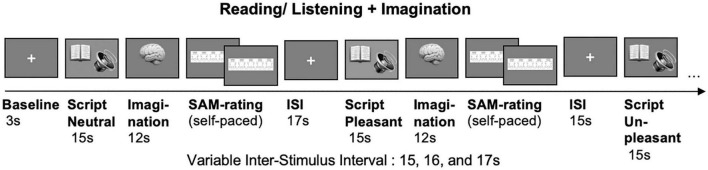
Imagination task. Unpleasant (U), neutral (N) and pleasant (P) scripts are presented for 15 s (listening/reading), followed by a 12 s imagination period and the self-paced Self-Assessment Manikin (SAM) rating. Variable inter-stimulus interval (ISI) of 15, 16, and 17 s.

In the silent reading + imagination task (see Figure 2), each script will be presented for 15 s followed by a 12 s imagination period. Each imagination will be followed by a SAM-rating. A variable inter-stimulus interval of 15, 16, and 17 s will be used.

The procedure in the listening + imagination task will be identical to silent reading, except that participants will now listen to audio files. Emotion order and reading/listening tasks will be counterbalanced between pairs. Audio files for the listening task will be read by male or female voice and will be counterbalanced, too.

#### 2.3.2. Experiment B—Dyadic synchrony

In experiment B, participants will only perform the reading/listening + imagination task, seated facing each other at a distance of 1.5 m. One partner, viewing the screen, will read out the scripts presented analogous to experiment A, the other partner (with the back to the screen) is listening. Then, both imagine the scene for 12 s and do individual SAM-ratings. After three runs (unpleasant, neutral, pleasant in counterbalanced order), positions are changed.

### 2.4. Data acquisition and pre-processing

Subjective rating data will be registered *via* Presentation Software (Version 23.1; Neurobehavioral Systems Inc., Berkeley, CA, USA) and logfiles will be further processed using IBM SPSS Statistics Version 29.0 (IBM Corp., Armonk, NY, USA) SPSS (Version 29.0, Armonk, NY, USA). ECG and EDA will be measured using EcgMove4^1^ and EdaMove4^2^ (movisens GmbH, Karlsruhe, Germany), respectively. For the continuous single channel ECG signal (sampling rate of 1024 Hz), a dry electrode chest belt associated with the EcgMove4 system will be used. An EDA sensor associated with the EdaMove4 system will be used to continuously measure EDA (sampling rate of 32 Hz) using a wrist-band and two disposable electrodes are attached to the hypothenar. The built-in activity sensor will register acceleration in three-dimension, angular rate (gyroscope), air pressure and temperature.

Raw sensor data will be read out, visualized and saved using the free and open software UnisensViewer (movisens GmbH, Karlsruhe, Germany), and will then be exported into MATLAB (The MathWorks Inc., Natick, MA, USA; raw ECG data and skin conductance level, SCL, for EDA). For subsequent analyses, ECG raw data will be digitally sampled down at 400 Hz and corrected for artifacts using the Autonomic Nervous System Laboratory [ANSLAB; (47)]. The software automatically detects R-wave-triggers to marker single heart beats; misplaced R-wave-triggers will be checked and corrected whenever they occur. The data will be converted into heart rate in beats per minute for every half-second bin of the sampling period and further analyzed with SPSS. The built-in activity sensor data (acceleration in three-dimension, angular rate, and temperature) will be analyzed together with EDA because of the potential influence of these variables on EDA. To control for possible effects of differences in the overall skin conductance level (SCL) on phasic SCL changes, SCL will be range-corrected per subject using all available data points during all experimental tasks. Both, SCL and heart rate (computed by converting RR-intervals into beats per minute) will be reduced into 1 sbins for synchrony analyses.

Electroencephalogram will be measured using two nine-channel B-Alert X-Series mobile-wireless EEG systems^3^ (BIOPAC Systems Inc., Goleta, CA, USA). Channel configuration includes F3, F4, C3, C4, P3, P4, Fz, Cz, and POz (monopolar configuration) with linked mastoids as reference. EEG data acquisition and pre-processing will be realized *via* Acq*Knowledge* Acquisition and Analysis Software (BIOPAC Systems Inc., Goleta, CA, USA; including high-pass and low-pass filtering at 0.1 Hz and 67 Hz, respectively).

### 2.5. Data analysis

Questionnaires and behavioral data will be imported from Presentation into SPSS (Version 29; IBM, Armonk, NY, USA).

To investigate the emotion effect, ANOVAs involving the within-subjects factor emotion (unpleasant vs. neutral vs. pleasant) will be conducted. Involving the additional between-subjects factor partner (partner 1 vs. partner 2), requires a total sample size of *N* = 42 to reveal medium size effects [obtained with G*Power, Version 3.1; alpha error probability = 0.05; power = 0.95; partial η^2^ = 0.06; (48)]. For theses analyses, heart rate will be extracted between 1 s prior to picture onset (baseline) and picture offset (3 s). Skin conductance change will be scored as the maximum response (between 1 and 3 s after picture onset). During the imagery reading/listening task, autonomic reactions will be determined by subtracting amplitudes in the first second prior to script presentation from response averages during the 15 s read and 12 s imagery periods.

For ERP analyses, data will be segmented starting 100 ms before (baseline) and 1.200 ms after stimulus onset for each sensor and means ERPs for each emotion category and subject are calculated [see (23)]. Time-windows and sensor sites for EPN and LPP analyses will be chosen following visual and statistical data inspection of individual averages for each valence category, using Randomization Graphical User interface toolbox (49).

For the picture viewing paradigm, each valence block is expected to last approximately 15 min, depending on the speed of the self-paced SAM-ratings. In the imagination task, a run including the three valences will take about 3.5 min, again depending on individual SAM-rating durations.

Thus, for synchrony analyses, signal streams will be synchronized for both subjects and then transformed in interval data. These time rows will be imported into SPSS for further analysis.

For this purpose, EEG data will be exported as MATLAB file (synchronized data for both individuals) and transformed *via* script into Brain Vision Analyzer (Version 2.2.2; Brain Vision, Morrisville, NC, USA) format to allow for artifact correction, filtering and further processing with dual electroencephalography (EEG) analysis pipeline [DEEP; (50)].

Moreover, correlational analyses and Actor–Partner Interdependence Models (APIM) are planned to analyze the influence of synchrony on subjective and interpersonal ratings (51, 52). Separate APIMs are planned for each measure (Heart rate, skin conductance, EEG) in the different tasks (independent variables) and subjective bonds-rating after the experiments (dependent variables).

### 2.6. Outcome and hypotheses

Primary research question is the successful implementation of the picture viewing and imagination paradigms from an individual setting into the two-person interaction, aiming at the replication of the enhanced attention to emotion effect [motivated attention; (30)] in subjective ratings (SAM), physiological (ECG, EDA) and neuronal (EEG) markers, and the development and testing of new data analysis strategies. Secondary outcome will be the influence of synchrony in physiological and neuronal activity during picture viewing and imagination on subjective emotion experience and interpersonal ratings after the experiment (WAI-SR).

#### 2.6.1. Emotion effect

In line with standard ratings and previous studies, we expect higher subjective arousal ratings for emotional, relative to neutral, pictures and scenes, and valence ratings unpleasant < < *cps*:*it* > *neutral* < /*cps*:*it* > < pleasant according to the biphasic model (30). During picture viewing, we expect emotionally arousing pictures to evoke more pronounced initial HR deceleration, whereas pleasant pictures will provoke higher post-orienting acceleration relative to unpleasant stimuli over time (30). Higher skin conductance levels are expected for emotionally arousing, relative to neutral pictures (30). In ERPs, emotional modulation of the EPN is expected between 150 and 350 ms post-stimulus onset (36). Over centro-parietal electrodes, elevated Late Positive Potentials (LPPs) for emotionally arousing, relative to neutral pictures, are expected, starting at about 400 ms post-stimulus onset (23, 24, 35).

During imagery, analogous reaction patterns are expected for heart rate and skin conductance [(29, 37, 38); for summary, see Ji et al. (39)]. Possibly, the LPP emotion effect might emerge over more posterior electrodes, compared to picture viewing (40), if it is not blunted at all during imagery (41).

#### 2.6.2. Influence of physiological and neuronal synchrony on subjective ratings

In the individual setting, physiological and neural responses reliably covary with subjective emotion ratings, especially for arousal (30, 53). Thus, higher synchrony in ECG, EDA, and EEG patterns during picture viewing and imagination should result in higher concordance during SAM-ratings, particularly in arousal ratings.

#### 2.6.3. Influence of physiological and neuronal synchrony on interpersonal ratings

Given that higher synchrony between individuals has been associated with more positive relationships (7, 17, 20), participants with higher synchrony in HR, EDA, and EEG during picture viewing and imagination tasks are expected to provide better interpersonal ratings after the experiment on the bonds-subscale (WAI-SR).

## 3. Discussion

The present study will provide an experimental approach to investigate interpersonal synchrony, using emotion-induction paradigms that are well-established in individual settings. The controlled experimental setting allows for the simulation of interactions from psychotherapy, and the establishment of research methods, which can later be translated into more naturalistic settings. The standardized emotion elicitation procedure and thus, controllability of an experiment, is a major strength for establishing synchrony methodology and understanding emotion processing in dyads. However, of course, it limits the generalization to real psychotherapy interactions. To increase ecological validity and to elicit more spontaneous emotional reactions, the dyadic interaction task as described by Roberts et al. (54) would be suited for subsequent experimental research. But this procedure is far less standardized and requires significantly more resources (e.g., topic identification, protocol), making it less suitable for the present study.

The study’s primary outcome will be the replication of the emotion effect in dyads, and, especially during interactions, an effect which is such a common finding in the individual setting with established methods (30). Maybe, altered attention to emotion will be observed in dyads, as reflected by different neural and physiological responses. One possible explanation would be the (implicit or explicit) use of emotion regulation strategies (55), e.g., attentional deployment or emotion suppression, when facing an interaction partner. For example, if participants were instructed to suppress emotional responses, increased skin conductance, but stronger heart rate deceleration have been observed, suggesting differential activation patterns during emotion suppression (56). Notably, participants showed no differences in subjective reports after affective suppression, and no sex differences were observed (56). Emotion regulation, e.g., attentional deployment, is also reflected by blunted LPP responses toward arousing stimuli (57). During interaction, both partner’s emotions (including empathy) and emotion regulation strategies are involved in a context-dependent manner (58). Keeping this in mind, using standard stimuli instead of individual, personal emotional experiences might prevent such strong empathy effects. Although the present study does not involve explicit emotion regulation instructions, the screening includes the Emotion Regulation Questionnaire by [Gross and John (59); German Version: (60)], and allows for including habitual use of cognitive reappraisal and expressive suppression as covariates.

Interestingly, Koole and Tschacher (7) also integrated emotion regulation as third level into their Interpersonal Synchrony (In-Sync) model of psychotherapy, which describes the therapeutic alliance to be “[…] grounded in the coupling of patient and therapist’s brains.” Along with the In-Sync model, the present study might complement level one, which is movement synchrony, with physiological and neural synchrony correlates, and complex cognition (level two) might be affected as well by the present emotion induction (and measured *via* subjective reports).

In line, the secondary outcomes include the relationship between physiological and neural synchrony and (1) subjective emotional experience, and (2) interpersonal relationship. Whilst it is very likely that high physiological and neural synchrony will go along with similar subjective ratings, at least on the arousal dimension (30, 53), there is some evidence for more positive relationships with higher synchrony (17, 20, 61); but this correlation has been mainly observed in parent-child and romantic partner interactions, and has been shown to grow with experience [(18); for review, see Koole et al. (7)]. Therefore, this relationship might be more pronounced in individuals who indicate being more familiar with each other in the beginning of the experiment. Notably, higher SCL synchrony has been associated with therapeutic bond during imagery, but not during application of cognitive-behavioral strategies (such as psychoeducation or identification of automatic conditions) suggesting that the association between synchrony and (therapeutic) relationship might also be task-dependent (61).

Fundamental understanding of interpersonal synchrony is critical to further examine interactions in the psychotherapy setting, and to potentially positively influence the therapeutic relationship through targeted interventions in a next step (e.g., neurofeedback, biofeedback). This approach is in line with the vision of translating basic bio-psychological research into disorder-independent clinical psychotherapy research—and back (3). Here, we focus on (neuro-) physiological correlates of emotion processing, which would mainly relate to the function domain of negative emotionality in mental disorders. Thus, research using and extending the present approach might identify dysfunctional (emotional) interaction patterns in sub-clinical and clinical samples from a *trans*-diagnostic perspective. Only recently, Saul et al. (62) proposed to not only investigate inter-brain synchrony in social anxiety disorder, but also to improve treatment *via* adding neurofeedback, pointing to fascinating new research perspectives. Moreover, as Koole and Tschacher (7) point out, the In-Sync model of psychotherapy provides the framework for training psychotherapists using feedback on movement and language synchrony in order to improve the therapeutic relationship. This might also include physiological and neural feedback strategies.

To conclude, the third outcome of the present study is to inspire further investigations, aiming at conducting psychotherapy research that is “going beyond self-report data” and which adds the interactional perspective.

## Ethics statement

The study protocol was reviewed and approved by the Ethics Committee of the University Medicine Greifswald (BB167/22). All participants were required to provide written informed consent to participate in the study.

## Author contributions

MH and JW: conceptualization and writing—original draft. MH, HM, JR, TK, and JW: methodology, programming, and analyses planning. JW: supervision and administration. All authors contributed to the article and approved the submitted version.

## Footnotes

1
https://docs.movisens.com/Sensors/EcgMove4/#welcome

2
https://docs.movisens.com/Sensors/EdaMove4/#welcome

3
https://www.biopac.com/wp-content/uploads/b-alert-x10-setup.pdf
