# Supplementary material for: Physiological and neural synchrony in emotional and neutral stimulus processing: A study protocol
Source: Front Psychiatry. 2023 Mar 30;14:1133760. doi: 10.3389/fpsyt.2023.1133760 (PMC10097964; doi:10.3389/fpsyt.2023.1133760)
Supplement: Supplementary file 1 [file Data_Sheet_1.PDF]

SCRIPTS (German)

Unangenehm

Ich wache auf. keuche. Rauch füllt meine Lungen.
Ich stolpere blind aus dem Bett. krache gegen einen Stuhl.
Das Feuer breitet sich schnell aus.
verbrennt meine Haut. als ich versuche zu fliehen.

Ich höre das Kreischen von Bremsen. schaue auf.
sehe. wie ein Auto meine Freundin erfasst.
Ihr Bein ist zerschmettert. eine Ader zerfetzt.
Blut pumpt auf die Straße.

Ich wache plötzlich in meinem Schlafsack auf.
Es ist total dunkel und ich fühle.
wie eine Schlange meine Beine hochgleitet.
Ich schreie. versuche aus dem Schlafsack zu kommen.

Neutral

Ich greife die letzte Socke aus dem Trockner.
werfe sie in den vollen Wäschekorb.
Ich rieche den frischen Geruch sauberer Wäsche. noch warm vom Trockner.
Ich hebe den Korb an.

Ich nehme die Lebensmittel aus dem Auto.
Ich hebe die Einkaufstüte hoch.
drücke sie fest gegen meine Brust und lehne mich vor.
um den Kofferraum zu schließen.

Ich fahre mit dem Kamm durch mein feuchtes Haar.
kontrolliere den Sitz meiner Kleidung.
Das Wasser läuft in den Abfluss.
Ich drehe den Wasserhahn zu.

Angenehm

Ein langer Kuss. Mein Körper reagiert erst langsam.
dann im heftigen Rhythmus. Ich bin atemlos.
meine Haut kribbelt. Ich fühle sanfte Hände mich berühren.
mein Rücken krümmt sich.

Ich habe gerade zehn Millionen Euro gewonnen!
Es ist unglaublich. aber wahr.
Ich habe das Gewinnerlos der Lotterie gekauft.
Wahnsinn. ich schreie und hüpfe vor Freude!

Die Zuschauer toben und ich springe auf
juble mit ihnen. als mein Team ein Tor schießt.
Wir haben den Rückstand aufgeholt und
gewonnen. Der Lärm ist ohrenbetäubend.

SCRIPTS (English)

Unpleasant

I wake up. gasping. smoke filling my lungs.
I stumble blindly out of bed. crash into a chair.
The fire spreads quickly.
burning my skin as I try to escape.

I hear the screech of brakes. look up.
see a car hit my girlfriend.
Her leg is shattered. a vein torn.
blood pumping on the road.

I suddenly wake up in my sleeping bag.
It's totally dark and I feel
a snake slithering up my legs.
I scream. trying to get out of the sleeping bag.

Neutral

I grab the last sock out of the dryer.
I throw it into the full laundry basket.
I smell the fresh scent of clean laundry. still warm from the dryer.
I lift the basket.

I take the groceries out of the car.
I lift the grocery bag.
press it firmly against my chest and lean forward.
to close the trunk.

I run the comb through my damp hair.
check the fit of my clothes.
The water is running down the drain.
I turn off the faucet.

Pleasant

A long kiss. My body reacts slowly at first.
then in a violent rhythm.
I am breathless. my skin tingles.
I feel gentle hands touching me. my back arches.

I've just won ten million euros!
It's unbelievable. but true.
I bought the winning lottery ticket.
Madness. I scream and jump for joy!

The crowd goes wild and I jump up.
cheer with them when my team scores a goal.
We made up for the deficit and won.
The noise is deafening.

PICTURE STIMULI

Unpleasant pictures

Category/ Short	Number	Valence	Arousal
Attack			
Pit Bull	1300	3.55	6.79
Soldier	6212	2.19	6.01
AimedGun	6260	2.44	6.93
Spider	1205	3.65	5.79
Gun	2811	2.17	6.9
CarTheft	6571	2.85	5.59
Mutilation			
Mutilation	3150	2.26	6.55
Burn victim	3102	1.4	6.58
Mutilation	3051	2.3	5.62
BatteredFem	3225	1.92	5.77
Mutilation	9253	2	5.53
DeadMan	9412	1.83	6.72
Accident/ disgust			
BloodyKiss	2352.2	2.09	6.25
Corpse	9490	3.6	5.57
SickKitty	9561	2.68	4.79
CarAccident	9908	2.34	6.63
Kids	9520	2.46	5.41
Vomit	9325	1.89	6.01
HeadlessBody	3001	1.62	6.64
Fire	8485	2.73	6.46
**Mean**		**2.3985**	**6.127**

Neutral pictures

Category/ Short	Number	Valence	Arousal
Objects			
Cow	1670	5.82	3.33
Boat	5390	5.13	2.95
Baskets	7041	4.99	2.6
Rug	7179	5.06	2.88
Pole	7161	4.98	2.98
Plate	7233	5.09	2.77
PicnicTable	7026	5.38	2.63
People			
Chess	2840	4.91	2.43
Man	7493	5.35	3.39
Man	2190	4.83	2.41
NeuMan	2102	5.16	3.03
Factoryworker	2393	4.87	2.93
Woman	2026	4.82	3.4
Nature/ buildings			
Window	7490	5.52	2.42
Flowers	5731	5.39	2.74
Farmland	5720	6.02	2.8
Shipyard	7036	4.88	3.32
Grain	5726	6.23	2.84
Building	7491	4.82	2.39
Bridge2	7547	5.21	3.18
**Mean**		**5.223**	**2.871**

Pleasant pictures

Category/ Short	Number	Valence	Arousal
Animals			
Puppies	1710	8.34	5.41
Seal	1440	8.19	4.61
People			
Children	2345	7.41	5.42
Olypic Athlete	75	7.01	5.33
Children	2158	7.13	5
Erotic			
EroticCouple	4659	6.87	6.93
EroticCouple	4658	6.62	6.47
Erotic Couple	52	7.21	6.12
EroticCouple	4693	6.16	6.57
EroticCouple	4694	6.69	6.42
EroticCouple	4698	6.5	6.72
EroticCouple	4697	6.22	6.62
Sports/ Adventure			
SkyDivers	5621	7.57	6.99
Surfer	57	7.16	5.5
Laughing Children	3	7.3	4.61
Happy Family	28	7.76	5.07
HangGlider	5626	6.71	6.1
Skier	8190	8.1	6.28
Bungee	8179	6.48	6.99
Rafting	61	6.86	6.26
**Mean**		**7.1145**	**5.971**

References

Bradley, M. M., & Lang, P. J. (2007a). Affective Norms for English Text (ANET): Affective ratings of text and instruction manual. (Tech. Rep. No. D-1). University of Florida, Gainesville, FL.

Lang, P.J., Bradley, M.M., Cuthbert, B.N., 2008. International Affective Picture System (IAPS): Affective ratings of pictures and instruction manual. Technical Report A-8. University of Florida, Gainesville, FL.

Wessa, M., Kanske, P., Neumeister, P., Bode, K., Heissler, J., & Schönfelder, S. 82010). EmoPics: Subjektive und psychophysiologische Evaluationen neuen Bildmaterials für die klinisch-bio-psychologische Forschung. *Zeitschrift für Klinische Psychologie und Psychotherapie*. S1/11, 77.
