# Supplementary material for: Physiological and neural synchrony in emotional and neutral stimulus processing: A study protocol
Source: Front Psychiatry. 2023 Mar 30;14:1133760. doi: 10.3389/fpsyt.2023.1133760 (PMC10097964; doi:10.3389/fpsyt.2023.1133760)
Supplement: Supplementary file 2 [file Data_Sheet_2.PDF]

WAI-SR

3. Ich glaube, mein Interaktionspartner/ meine Interaktionspartnerin mag mich.
5. Mein Interaktionspartner/ meine Interaktionspartnerin und ich achten einander.
7. Ich spüre, dass mein Interaktionspartner/ meine Interaktionspartnerin mich schätzt.
9. Ich spüre, dass mein Interaktionspartner/ meine Interaktionspartnerin auch dann zu mir steht, wenn ich etwas tue, dass er/ sie nicht gutheißt.

3. I believe my interaction partner likes me.
5. My interaction partner and I respect each other.
7. I feel that my interaction partner appreciates me.
9. I feel my interaction partner cares about me even when I do things that he/ she does not approve of.
